# Mineral Concentration in Spring Wheat Grain Under Organic, Integrated, and Conventional Farming Systems and Their Alterations During Processing

**DOI:** 10.3390/plants14071003

**Published:** 2025-03-23

**Authors:** Katarzyna Wysocka, Grażyna Cacak-Pietrzak, Tomasz Sosulski

**Affiliations:** 1 Department of Food Technology and Assessment, Institute of Food Sciences, Warsaw University of Life Sciences (SGGW), Nowoursynowska 159C Street, 02-776 Warsaw, Poland; 2 Department of Agricultural and Environmental Chemistry, Institute of Agriculture, Warsaw University of Life Sciences (SGGW), Nowoursynowska 159 Street, 02-776 Warsaw, Poland; tomasz_sosulski@sggw.edu.pl

**Keywords:** wheat grain, flour, bran, bread, cultivars, macro- and micronutrients, mineral composition, nutritional value, farming system

## Abstract

Common wheat (*Triticum aestivum* L.) is a key cereal in the global economy, providing essential nutrients for human and animal health. The European Union promotes less intensive farming systems as part of its sustainable development strategy. This study aimed to evaluate the impact of different farming systems on the concentration of macronutrients—potassium, phosphorus, magnesium, and calcium (K, P, Mg, and Ca)— and micronutrients—iron, zinc, manganese, and copper (Fe, Zn, Mn, and Cu)—in wheat grain, as well as the effect of grain processing on the distribution of these nutrients in bran, flour, and bread. This study included four spring wheat cultivars (Harenda, Kandela, Mandaryna, and Serenada) grown under organic (ORG), integrated (INT), and conventional (CONV) systems at the Osiny Experimental Station (Poland; 51°27′ N; 22°2′ E) between 2019 and 2021. The P concentration was determined using the colorimetric method, while the other nutrients were analyzed by atomic absorption spectrometry (AAS). The grain from the CONV system exhibited higher macronutrients concentrations, whereas grain from less intensive systems had higher micronutrients concentrations, except for Fe. The Fe concentration in grain from the INT and CONV systems was comparable. An interaction effect between cultivars and farming systems on P, Ca, Mn, Zn, and Cu concentrations in the grain was observed. In all research material, the highest concentrations of minerals were found in bran, followed by grain, with the lowest concentrations observed in flour and bread.

## 1. Introduction

Common wheat (*Triticum aestivum* L.) is one of the most widely cultivated cereals, playing a key role in the global food system [1]. Global wheat production has shown an upward trend in recent years, with Asia and Europe dominating its production. Major producers of this cereal include countries such as China, India, Russia, the United States, Canada, France, and Pakistan. In Poland, wheat production in the 2022/2023 season amounted to 12.9 million tons [2]. In many countries, wheat constitutes a dietary staple, particularly in regions with high consumption of cereal-based products [3]. It is widely used for producing foods such as pasta, groats, flakes, and biscuits, but most importantly for baking bread [4], which is considered globally an essential food for human nutrition [5]. Additionally, wheat is an industrial raw material used to prepare alcoholic beverages, starch, straw, and animal feed [6,7,8].

Wheat is a fundamental food ingredient that offers a variety of beneficial nutritional components, including carbohydrates, protein, dietary fiber, fat, minerals, B vitamins, and polyphenol compounds [9]. The minerals in wheat are primarily located in the germ, husk, and aleurone layer [10,11]. As a result, wheat bran is richer in these minerals compared to refined flour made from milling wheat grains [12,13]. These minerals are categorized into macronutrients (such as potassium, phosphorus, sodium, calcium, and magnesium), which are found in larger quantities, and micronutrients, also called trace elements (including zinc, copper, molybdenum, iron, boron, and selenium), which are present in smaller amounts [14]. Both types of minerals are crucial for the proper functioning of human and animal bodies. They play vital roles in several biological processes, including energy storage and utilization, protein metabolism, reducing inflammation, transporting oxygen, regulating heart rhythm, supporting bone metabolism, and enhancing immune function [15,16]. A deficiency in specific minerals can lead to various chronic diseases [14,17,18].

The concentration of macro- and micronutrients in wheat grain depends on environmental factors (soil and weather conditions), genotype (cultivar), and agrotechnology [19,20,21,22,23,24,25]. Among these factors, agrotechnology, including the farming system, plays a crucial role as it differentiates soil tillage methods, fertilization, and plant protection practices [20,21,25,26,27,28].

In light of the above, this study aimed to determine the effect of the applied farming systems on the concentration of minerals (macro- and micronutrients) in the grain of selected spring wheat cultivars. The research hypothesis assumed that the mineral concentration in the grain depends on the applied agronomic practices, the wheat cultivar, and the interaction between these factors. Furthermore, the scope of the study was extended to include an assessment of mineral concentrations in byproducts obtained from grain processing (bran, flour, and bread) to show how the concentration of these components changes at successive processing stages (milling and baking) and determine their final content in the final product, i.e., bread.

## 2. Results

### 2.1. Concentrations of Macro- and Micronutrients in Spring Wheat Grain

The macronutrients concentration in grain ranged from 4.31 to 5.20 g K kg^−1^, from 2.48 to 4.09 g P kg^−1^, from 1.05 to 1.30 g Mg kg^−1^ and from 0.26 to 0.67 g Ca kg^−1^ dry matter (d.m.), respectively (Table S1). The macronutrients concentration in the grain was significantly influenced by the harvest year (Table 1). The highest concentration of K and P was found in the grain harvested in 2020 (mean 5.04 g K kg^−1^ d.m. and 3.47 g P kg^−1^ d.m., respectively), while the lowest nutrients concentration was observed in grain from 2021 (mean 4.67 g K kg^−1^ d.m. and 3.17 g P kg^−1^ d.m., respectively). The grain harvested in 2021 had significantly higher Mg concentration (mean 1.19 g Mg kg^−1^ d.m.) than in 2019 and 2020 (mean 1.14 g Mg kg^−1^ d.m.). However, the highest Ca concentration was observed in grain from 2019 (mean 0.48 g Ca kg^−1^ d.m.), which was significantly higher than in grain from 2020 (mean 0.39 g Ca kg^−1^ d.m.).

The cultivar factor had a significant effect only for P and Ca concentrations in the grain. The Serenada cultivar had the highest P concentration (mean 3.52 g P kg^−1^ d.m.), significantly higher than the grain of the Mandaryna cultivar (mean 3.03 g P kg^−1^ d.m.). Asignificantly lower Ca concentration was detected in the grain of the Serenada cultivar (mean 0.37 g Ca kg^−1^ d.m.) compared to the other wheat cultivars.

The farming system significantly differentiates the concentration of Ca only. The grain from the CONV system exhibited the highest significant Ca concentration (mean 0.48 g Ca kg^−1^ d.m.), while the grain from the ORG system had the lowest significant Ca concentration (mean 0.38 g Ca kg^−1^ d.m.).

**Figure 1:**
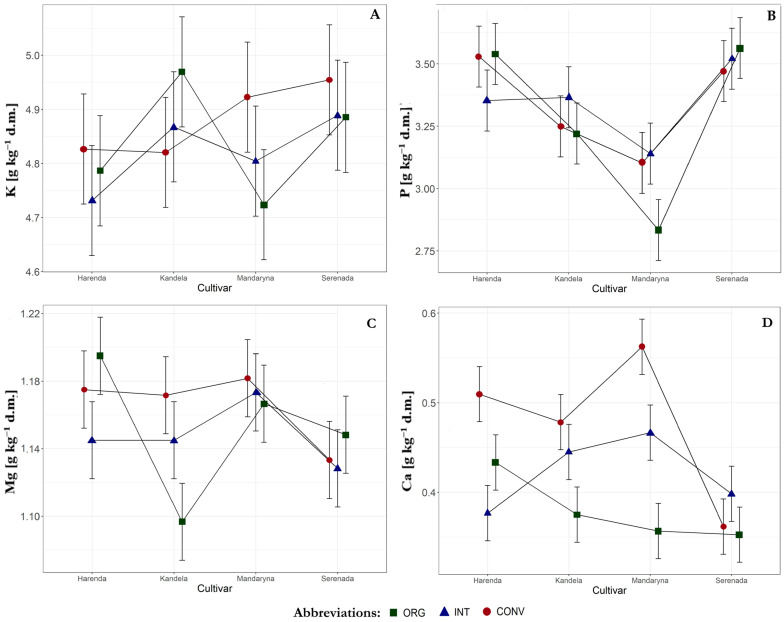
The interaction effects of cultivar and farming system on macronutrient concentration in the grain: (**A**) K, (**B**) P, (**C**) Mg, (**D**) Ca. Abbreviations: ORG—organic, INT—integrated, CONV—conventional, K—potassium, P—phosphorus, Mg—magnesium, Ca—calcium.

We identified interactions between cultivars and farming systems concerning the concentrations of P and Ca in the grain (Figure 1B,D). The highest P concentrations were observed in grains from systems with less intensive agronomic practices, specifically the ORG system (the cultivars Harenda and Serenada) and the INT system (the cultivars Kandela and Mandaryna). In contrast, the application of intensive agrotechnology (the CONV system) resulted in higher Ca concentrations in the grain of most wheat cultivars, except for the cultivar Serenada. To obtain grain with high concentrations of the analyzed macronutrients, the CONV system is recommended for the cultivars Kandela and Mandaryna. In contrast, the CONV and ORG systems are suitable for the cultivar Harenda. For the cultivar Serenada, the ORG system had a beneficial effect on P and Mg concentrations in the grain; however, it is worth emphasizing that this cultivar showed the least variation in macronutrients concentration across different farming systems.

The micronutrienst concentration in the grain samples ranged from 22.18 to 72.58 mg Fe kg^−1^, from 13.44 to 41.13 mg Mn kg^−1^, from 21.59 to 51.24 mg Zn kg^−1^, and from 1.09 to 4.18 mg Cu kg^−1^ dry matter (d.m.), respectively (Table S1). Except for Cu, the micronutrients concentration in the grain was significantly influenced by the harvest year (Table 2). Significantly higher Fe concentration was observed in wheat grain harvested in 2021 than in 2020 and 2019. The highest significant Mn and Zn concentration was observed in grain harvested in 2020.

The cultivar factor had a significant effect only on Mn and Zn concentrations (Table 2). The grain of the Serenada cultivar had the highest Mn and Zn concentrations (mean 25.51 mg Mn kg^−1^ d.m., and 34.81 mg Zn kg^−1^ d.m.), which was significantly higher than that of the Mandaryna and Kandela cultivars (mean: 21.59 and 22.28 mg Mn kg^−1^ d.m. and 27.05 and 26.59 mg Zn kg^−1^ d.m., respectively).

The farming system significantly differentiates micronutrients, except for Fe (Table 2). The highest Mn concentration was found in grain from the INT system (mean 27.26 mg Mn kg^−1^ d.m.), while the lowest significant Mn concentration was observed in grain from the ORG system (mean 18.82 mg Mn kg^−1^ d.m.). The grain from the ORG system exhibited the highest Zn concentration (mean 31.11 mg Zn kg^−1^ d.m.), which was significantly higher than the grain from the CONV system (mean 27.02 mg Zn kg^−1^ d.m.). The highest significant Cu concentration was observed in grain from the ORG system (mean 3.46 mg Cu kg^−1^ d.m.), whereas Cu concentration in grain from the CONV system was nearly half as low (mean 1.85 mg Cu kg^−1^ d.m.).

**Figure 2:**
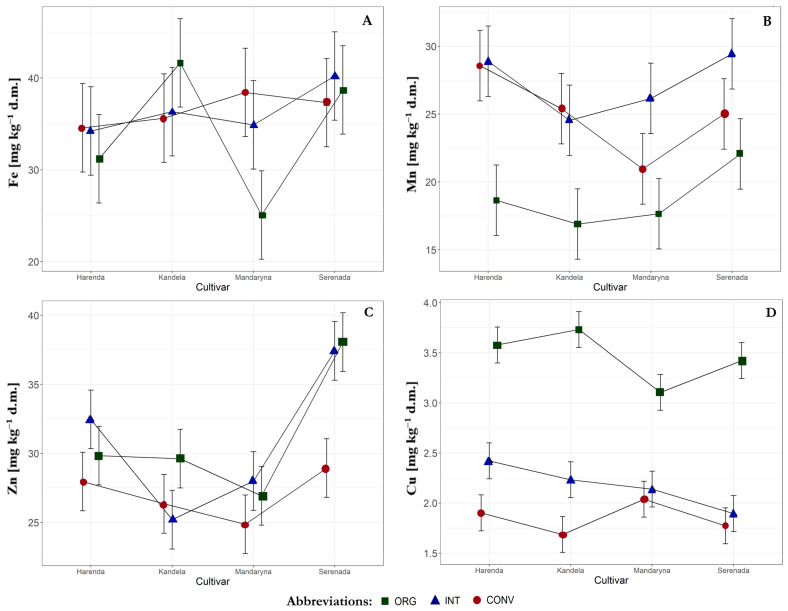
The interaction effects of cultivar and farming system on micronutrient concentration in the grain: (**A**) Fe, (**B**) Mn, (**C**) Zn, (**D**) Cu. Abbreviations: ORG—organic, INT—integrated, CONV—conventional, Fe—iron, Zn—zinc, Mn—manganese, Cu—copper.

We identified interactions between cultivars and farming systems concerning the grain concentrations of Mn, Zn, and Cu (Figure 2B–D). The highest Mn concentration was found in grain from the INT system (the cultivars Harenda, Mandaryna, and Serenada) and the CONV system (the cultivar Kandela). In contrast, the least favorable effect on the Mn concentration in the grain of all wheat cultivars was observed under the ORG system. The highest Zn concentration was found in grains from less intensive farming systems, specifically the ORG system (the cultivars Kandela and Serenada) and the INT system (the cultivars Harenda and Mandaryna). All tested wheat cultivars showed the highest Cu concentrations in grains from the ORG system, while the lowest concentrations were observed in grains from the CONV system. To obtain grain with high concentrations of the studied micronutrients, it is generally recommended to cultivate in systems with less intensive agronomy (ORG and INT). Particularly favorable responses to cultivation in these systems were observed for the cultivar Serenada.

Significant correlations between the analyzed minerals in wheat grain were observed (Figures S1–S3). In the CONV system, significant positive correlations in grain were found between Mn and Zn (r = 0.73), Mg and Cu (r = 0.65), K and P (r = 0.64), P and Mg (r = 0.58), and Mg and Ca (r = 0.58). Negative correlations in the CONV system were observed between K and Fe (r = −0.78), P and Fe (r = −0.65), and Ca and Zn (r = −0.64) (Figure S1). In the INT system (Figure S2), significant positive correlations were found between Mn and Zn (r = 0.79), Mg and Cu (r = 0.67), while a negative correlation was found between Ca and Mn (r = −0.68). In the ORG system (Figure S3), a significant positive correlation was found between P and Zn (r = 0.75).

### 2.2. Concentrations of Macro- and Micronutrients in Wheat Grain and Their Byproducts

The macronutrients concentration varied significantly depending on the analyzed research material, as presented in Table 3. The highest significant concentrations of individual macronutrients were observed in bran (BN), followed by lower concentrations in grain (GR), and the lowest concentrations in flour (FR) and bread (BD), which, in turn, did not differ significantly in terms of macronutrient concentrations. Ca was the exception, where the concentrations in GR and BD were not significantly different. The concentration of K in BN was nearly twice as high as in GR, Ca and P were over twice as high, and Mg was approximately three times higher. Among all measured macronutrients in wheat grain and its byproducts, K exhibited the highest concentration.

Micronutrients concentration, similar to the analyzed macronutrients, varied significantly depending on the investigated research material, as shown in Table 4. The highest significant concentrations of the analyzed micronutrients were observed in BN, which were generally approximately three times higher compared to their concentrations in GR. The concentrations of all analyzed micronutrients were higher in BD than in FR, with statistically significant differences observed for Fe and Cu. The concentrations of Fe and Cu in BD were approximately twice as high as in FR, whereas the concentration of Mn in BD was slightly higher than in FR. The Cu concentration in GR was similar to that in BD. Among the analyzed micronutrients, the highest concentration in GR and its byproducts were recorded for Fe and Zn, while the lowest concentration was observed for Cu.

## 3. Discussion

Wheat is a fundamental raw material in the food industry, primarily used for flour production, which serves as the base ingredient for bread, pasta, confectionery, and various other food products [3,29,30]. In addition to its role as a staple food, wheat provides essential macro- and micronutrients, the concentration of which, as indicated by the literature data [20,21,22,24,25,31,32,33], depends on genetic factors (cultivar), environmental conditions, agricultural practices, and complex interactions between these factors.

Weather conditions significantly influence the concentration of macro- and micronutrients in cereal grains, as temperature, precipitation, and sunlight affect plant nutrient uptake. Environmental variability impacts both physiological processes in plants and the chemical properties of the soil, ultimately determining the yield’s final quality and mineral composition [34]. Whether moderate or severe, drought reduces the uptake of both macro- and micronutrients, such as N, P, K, Ca, Mg, Fe, S, Zn, and Cu [35].

These findings are further supported by our research, which demonstrates that weather conditions during individual growing seasons had the most significant impact on the mineral concentration in wheat grain among the studied factors. The harvest year significantly affected the concentration of most analyzed minerals, except for Cu. This can be attributed to the relatively stable presence of Cu in the soil, as it is less prone to leaching by rainfall compared to other elements, such as nitrogen (N) and potassium (K) [36,37]. However, drought conditions limit the bioavailability of Cu to plants, a trend confirmed by our study results. A slightly lower Cu concentration was observed in wheat grain from the 2019 harvest, which correlated with lower total precipitation compared to other growing seasons [38]. In the analyzed wheat grain samples, K and P were the most abundant macronutrients, which aligns with findings reported in the literature [20,21,39]. In our study, K accounted for an average of 50% of the total macronutrient concentration in wheat grain, while P constituted approximately 34%. The highest statistically significant concentrations of K and P were observed in the grain from 2020, which may be attributed to the lower wheat yield recorded that year. These results were reported in an earlier publication [38]. A negative correlation between grain yield and mineral concentration has also been suggested by Golea et al. [40], who stated that higher yields may be associated with lower macro- and micronutrients concentrations due to the so-called dilution effect. This phenomenon occurs when increased biomass production leads to a more extensive distribution of mineral nutrients across a larger volume of plant material, ultimately reducing their concentration in individual grains.

The significant influence of weather conditions on the mineral composition of wheat grain, as demonstrated in our study, is further supported by Caldelas et al. [39]. However, Jaskulska et al. [20] did not confirm a substantial impact of climatic factors on the concentration of macronutrients, such as K, P, Mg, and Ca, in wheat grain. Similarly, Hussain et al. [41], in a six-year study on organically grown wheat genotypes, did not observe a consistent trend in the effect of environmental conditions on macronutrients accumulation in the wheat grain.

The literature on the subject also indicates cultivar differences in the concentration of macro- and micronutrients in wheat grain [23,25,39,41,42]. This was partially confirmed in our research. A statistically significant effect of the cultivar factor was observed for four of the eight analyzed minerals: P, Ca, Mn, and Zn. In contrast, the study by Caldelas et al. [39], conducted on grain from 12 wheat cultivars, demonstrated a significant influence of the cultivar on the concentration of all analyzed macroelements, including K, P, Mg, Ca, and S. According to these authors, the cultivar was the most critical factor affecting mineral concentration in the grain, followed by the weather conditions. Similarly, Rachoń et al. [22] reported interspecies variation in wheat, indicating higher concentrations of K, P, Mg, and Ca in the grain of *T. monococcum*, *T. durum*, and *T. spelta* compared to *T. aestivum*.

In the present study, the farming system significantly impacted the concentration of only one micronutrient: Ca. In the case of Ca, increased production intensity resulted in higher concentrations of this macronutrient in the grain. Some studies have shown that higher nitrogen (N) fertilizer application rates increase the P and K concentration in wheat grain [27,28], which was not confirmed by our findings. In our study, higher concentrations were generally found in the grain from less intensive systems for micronutrients. The exception was Mn and Fe, where no statistically significant differences were observed. Similarly, Caldelas et al. [39] did not find any impact of differentiated nitrogen fertilization on the concentration of these minerals in wheat grain.

The increased concentration of some micronutrients, such as Cu, in the grain from organic systems may be attributed to the fact that organic fertilizers, such as manure and compost, often contain higher levels of Cu than mineral fertilizers [43]. Furthermore, in CONV systems, the high nitrogen (N) doses can antagonistically affect the uptake of certain elements, including Cu, thereby reducing its absorption by plants [44,45]. Ryan et al. [46] noted copper dilution at higher grain yields in CONV systems. It is important to emphasize that, in our study, Cu concentration in wheat grain was significantly influenced only by the farming system. Less intensive farming systems led to increased Cu concentration in the grain, especially in the ORG system.

In contrast, the concentration of micronutrients such as Mn and Zn was affected by all experimental factors. Ryan et al. [46] observed only minor differences in N, K, Mg, Ca, S, and Fe concentrations in wheat grain between CONV and ORG systems. Their study indicated that conventional wheat grain had lower Zn and Cu concentrations but higher Mn and P concentrations than organic wheat grain. Similar trends for Zn and Cu concentrations were observed in our study.

Wheat is one of the significant cereals processed for human consumption [30,47]. The human body cannot synthesize macro- and micronutrients, which must come from the diet or supplements, and their deficiency can lead to serious health issues [48]. Cereal-based products, especially bread, are an essential mineral source in the daily diet [41]. Bread is a good source of many minerals. Still, it is necessary to note that their concentration primarily depends on the type of flour used, which is related to the degree of processing of the grain and its mineral concentration in other recipe ingredients. Whole wheat bread contains significant concentrations of K, Mg, P, Ca, and Fe. In contrast, bread made from refined flour has a considerably lower mineral concentration, primarily due to the uneven distribution of minerals in different anatomical parts of the grain, such as the husk, germ, and endosperm [10,12,41].

Minerals are primarily located in the germ, husk, and aleurone layer of the wheat grain [10]. In our experiment, the mineral concentration in byproducts differed depending on the wheat grain processing method (milling and baking). The highest concentrations of both macro- and micronutrients were found in bran. Stevenson et al. [49] also indicate higher mineral concentrations in bran. When we compared the mineral concentrations in flour and bread, our findings showed higher levels of both macro- and micronutrients in bread made from wholemeal flour compared to refined flour, which can be attributed to the use of baker’s yeast in the recipe [50]. As Sun et al. [51] reported, baker’s yeast is a source of micronutrients such as Cu, Fe, and Mn.

Wheat bread from our experiment was particularly rich in K and P. These macronutrients are commonly present in food, which makes deficiencies relatively rare. However, due to their lower concentration in food products and minor bodily requirements, micronutrients are more susceptible to deficiencies, especially in poorly balanced diets or absorption disorders [52].

Among macronutrients, K is a critical electrolyte in the human body, essential for proper nerve function, muscle contractions (including heart muscles), blood pressure regulation, and maintaining water–electrolyte balance [53]. P plays essential roles in various tissues throughout the body, serving as an integral component of hydroxyapatite in bones and as a substrate for ATP biosynthesis [54]. Mg is a key enzyme cofactor involved in ATP production, protein synthesis, nerve conduction, muscle contractions, and blood pressure regulation, and Ca is a primary structural component of bone tissue, contributing to skeletal strength and integrity [55].

Micronutrients are essential components of the human diet, required in relatively small amounts. However, it is estimated that approximately 3 billion people worldwide suffer from micronutrient deficiencies, leading to significant health consequences [56,57,58]. Unfortunately, wheat naturally contains low levels of Zn and Fe [57,58], which, according to the WHO data [59], are among the most common nutrient deficiencies affecting the global population.

Deficiencies of these minerals are the most prevalent in developing countries, where diets are typically inadequate in quantity and quality. In other regions, these deficiencies are primarily associated with specific health conditions rather than insufficient dietary intake [60].

Fe is crucial in various metabolic processes, including oxygen transport and DNA synthesis [61]. Fe accounted for more than 50% of the total micronutrient concentration in the analyzed bread. Zn is essential for multiple physiological functions, including cell growth and development, metabolism, and the proper functioning of the cognitive, reproductive, and immune systems [62]. Mn plays a vital role in biochemical processes, such as enzyme activation, and is involved in carbohydrate, lipid, and protein metabolism and cellular protection against oxidative stress [63]. Additionally, Cu is essential for energy production, connective tissue formation, and defense against oxidative damage [64,65].

According to the EFSA recommendations [66], the consumption of 100 g of the analyzed bread covered a certain percentage of the daily requirement for key macronutrients for adults: 33% P; 11–13% Mg; and 8% K and 3% Cu for men and women, respectively. Micronutrients covered 22% of the daily Fe requirement for adult men, while in premenopausal women, it covered 15% and, for postmenopausal women, 22%. Mn was covered at 19% regardless of gender, while Cu was covered at approximately 15%. The lowest coverage was observed for Zn, which accounted for about 12% of the daily requirement for adult men and postmenopausal women, whereas for premenopausal women, it covered approximately 8%.

## 4. Materials and Methods

### 4.1. Research Material

**Figure 3:**
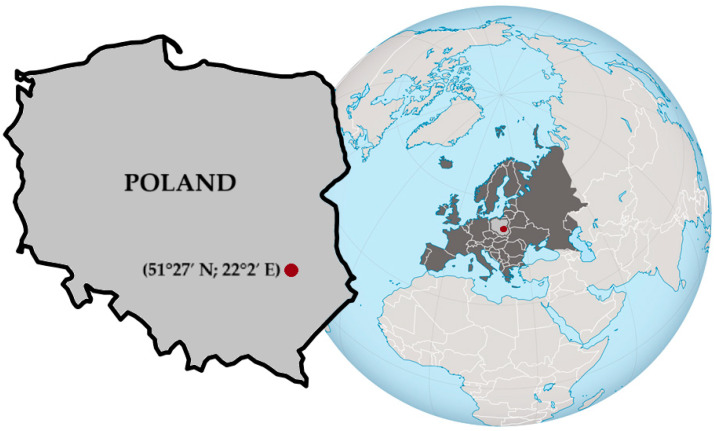
Field experiment location. ● Osiny (Poland; 51°27′ N; 22°2′ E).

The study material consisted of the wheat grain of four cultivars—Harenda, Kandela, Mandaryna, and Serenada—grown in three different farming systems—organic (ORG), integrated (INT), and conventional (CONV)—at the Experimental Station of IUNG-PIB in Osiny (51°27′ N; 22°2′ E) in Poland (Figure 3) between 2019 and 2021. Byproducts from milling wheat grains (flour and bran) and bread baked from the obtained flour were also analyzed. The comprehensive methodology of the field trial (physical and chemical soil properties before the experiment and the application of fertilizers and plant protection chemicals, with a description of the meteorological conditions in the growing season, were presented in a previous publication [38]. The grain was milled in a two-lane Quadrumat Senior mill (Brabender Instruments, Duisburg, Germany) in accordance with AACC Method No. 26-50.01 [67]. After milling, a milling balance was prepared, and then flour mixtures with 70% extract were prepared. The dough recipe consisted of 500 g of wheat flour, 15.0 g of yeast (Lesaffre Polska S.A., Wolczyn, Poland), 7.5 g of table salt (Klodawa S.A., Klodawa, Poland), and water in the amount needed to obtain a dough with a consistency of 350 FU. After baking, 250 g bread samples were dried and then ground in a laboratory mill (A11, IKA Works GmbH and Co., Staufen, Germany) to particle sizes below 1.0 mm. The methodology of grain milling and the bread-baking processes were presented in detail in another previous publication [50].

### 4.2. Methods—Macro- and Micronutrient Determination

First 1 g of homogenized material samples, grain, and grain byproducts (flour, bran and bread) were subjected to wet mineralization in a mixture of 65% nitric acid (HNO_3_) and 70% perchlorine acid (HClO_4_) in a 4:1 ratio, using a digestion unit (Velp Scientifica, DK 20 Heating Digester, Usmate (MB), Italy), and then the concentration of macro- (K, Mg, and Ca) and micronutrients (Fe, Mn, Zn, and Cu) were determined using atomic absorption spectrophotometry (AAS) (Thermo Scientific iCE 3000 Series, AA Spectrometer, Cambridge, UK). The wavelengths of determined elements were as follows: K, 766.5 nm; Mg, 285.2 nm; Ca, 422.7 nm; Fe, 248.3 nm; Mn, 279.5 nm; Zn, 312.9 nm; and Cu, 324.8 nm. The phosphorus (P) content in the samples was determined by the colorimetric method, using a GENESYS 10S UV-VIS (ultraviolet and visible light region) spectrophotometer (Thermo Scientific, Waltham, MA, USA), according to the vanadate–molybdate method (wavelength: 470 nm).

### 4.3. Statistical Analyses

All measurements were made in three replicates. To compare the influence of the studied factors, harvest year (*n* = 3), cultivar (*n* = 4), and farming system (*n* = 3), and their interaction effects on the mineral composition of the wheat grain, the analysis of variance ANOVA was applied, and the mean differences were evaluated using Tukey’s test at the significance level of α = 0.05. The differences in the concentration of analyzed minerals for wheat grain and its byproducts were assessed using one-way ANOVA. Tukey’s test was applied as the post hoc test for these two types of variance analysis. The assumptions for applying ANOVA, including the normality of distribution and homogeneity of variances, were verified and found to be satisfactory. The relationship between all study traits was evaluated (separately for each type of farming system) using the Pearson correlation coefficient. The statistical analyses were performed using R 4.2.1 software.

## 5. Conclusions

The results of this study indicate that the applied farming system may influence the concentration of both macro- and micronutrients in wheat grain. Our analysis reveals a slightly higher concentration of macronutrients in grain derived from the CONV system. In comparison, a higher concentration of micronutrients was found in grain from less intensive systems (ORG and INT), except for Fe, which was slightly higher in CONV grain. Among the analyzed micronutrients, the farming system significantly affected the concentration of Mn, Zn, and Cu in wheat grain, whereas, among macronutrients, this effect was observed only for Ca. Furthermore, this study demonstrates that the cultivars responded differently to the applied farming systems in terms of P, Ca, Mn, Zn, and Cu concentrations in the grain.

Moreover, this study demonstrates that the concentration of both macro- and micronutrients in wheat grain byproducts differs significantly from their initial levels in the grain. Bran, a byproduct of wheat milling, exhibited the highest concentration of nutrients. In contrast, their concentration in refined flours was several times lower, which resulted from the uneven distribution of these nutrients in different anatomical parts of the wheat kernel. Our findings suggest that wheat bread produced from refined flour with the addition of baker’s yeast constitutes an excellent source of P. For instance, the consumption of a 100 g portion of this bread covers more than one-third of the daily P intake recommended by the EFSA for an adult. The contribution of a 100 g portion of this bread to meeting the dietary requirements for analyzed macro- and micronutrients ranged from 3% (Cu) to 33% (P). However, since bread is a staple food in the daily diet, its role in providing essential nutrients to the human body should be considered significant.

## Figures and Tables

**Table 1:** The macronutrients concentration in the wheat grain [g kg^−1^ d.m.] based on the harvest year, cultivar, and farming system.

Source of Variation	K	P	Mg	Ca
**Year**	**	**	**	**
2019	4.84 ^b^ ± 0.25	3.33 ^ab^± 0.36	1.14 ^a^ ± 0.07	0.48 ^b^ ± 0.10
2020	5.04 ^c^ ± 0.11	3.47 ^b^ ± 0.32	1.14 ^a^ ± 0.03	0.39 ^a^ ± 0.10
2021	4.67 ^a^ ± 0.18	3.17 ^a^ ± 0.31	1.19 ^b^ ± 0.05	0.41 ^ab^ ± 0.06
**Cultivar**	**n.s.**	**	**n.s.**	**
Harenda	4.78 ^a^ ± 0.18	3.47 ^b^ ± 0.19	1.17 ^a^ ± 0.05	0.44 ^b^ ± 0.11
Kandela	4.89 ^a^ ± 0.30	3.28 ^b^± 0.33	1.14 ^a^ ± 0.08	0.43 ^b^ ± 0.08
Mandaryna	4.82 ^a^ ± 0.29	3.03 ^a^ ± 0.28	1.17 ^a^ ± 0.04	0.46 ^b^ ± 0.11
Serenada	4.91 ^a^ ± 0.15	3.52 ^b^ ± 0.36	1.14 ^a^ ± 0.05	0.37 ^a^ ± 0.04
**Farming system**	**n.s.**	**n.s.**	**n.s.**	**
ORG	4.84 ^a^ ± 0.26	3.29 ^a^ ± 0.38	1.15 ^a^ ± 0.23	0.38 ^a^ ± 0.04
INT	4.82 ^a^ ± 0.21	3.34 ^a^ ± 0.31	1.15 ^a^ ± 0.06	0.42 ^b^ ± 0.08
CONV	4.88 ^a^ ± 0.25	3.34 ^a^ ± 0.36	1.17 ^a^ ± 0.05	0.48 ^c^ ± 0.12

The data are presented as means with their respective standard deviations. Abbreviations: ** denotes significance, where different letters on the top of data ^a–c^ indicate significant differences at the α = 0.05 level between means according to Tukey’s test. n.s.—not significant, ORG—organic, INT—integrated, CONV—conventional, K—potassium, P—phosphorus, Mg—magnesium, Ca—calcium.

**Table 2:** The micronutrients concentration in wheat grain [mg kg^−1^ d.m.] based on the harvest year, cultivar, and farming system.

Source of Variation	Fe	Mn	Zn	Cu
**Year**	**	**	**	**n.s.**
2019	32.40 ^a^ ± 4.50	20.12 ^a^ ± 3.08	25.17 ^a^ ± 3.10	2.37 ^a^ ± 0.71
2020	28.35 ^a^ ± 6.54	29.26 ^b^ ± 8.34	33.33 ^b^ ± 8.17	2.60 ^a^ ± 0.98
2021	46.29 ^b^ ± 13.07	21.69 ^a^ ± 5.71	30.41 ^b^ ± 3.53	2.51 ^a^ ± 0.80
**Cultivar**	**n.s.**	**	**	**n.s.**
Harenda	33.33 ^a^ ± 4.08	25.38 ^b^ ± 8.27	30.09 ^b^ ± 4.03	2.63 ^a^ ± 0.75
Kandela	37.87 ^a^ ± 17.78	22.28 ^a^ ± 6.47	27.05 ^a^ ± 3.01	2.55 ^a^ ± 0.97
Mandaryna	32.79 ^a^ ± 13.96	21.59 ^a^ ± 6.67	26.59 ^a^ ± 2.26	2.43 ^a^ ± 0.73
Serenada	38.74 ^a^ ± 2.99	25.51 ^b^ ± 7.10	34.81 ^c^ ± 9.62	2.36 ^a^ ± 0.89
**Farming system**	**n.s.**	**	**	**
ORG	34.15 ^a^ ± 14.15	18.82 ^a^ ± 2.84	31.11 ^b^ ± 5.69	3.46 ^c^ ± 0.54
INT	36.42 ^a^ ± 9.01	27.26 ^b^ ± 6.53	30.77 ^b^ ± 7.81	2.17 ^b^ ± 0.45
CONV	36.49 ^a^ ± 12.62	24.99 ^b^ ± 8.38	27.02 ^a^ ± 4.18	1.85 ^a^ ± 0.31

The data are presented as means with their respective standard deviations. Abbreviations: ** denotes significance, where different letters on the top of data ^a–c^ indicate significant differences at the α = 0.05 level between means according to Tukey’s test. n.s.—not significant, ORG—organic, INT—integrated, CONV—conventional, Fe—iron, Mn—manganese, Zn—zinc, Cu—copper.

**Table 3:** Macronutrients concentration in wheat grain and grain byproducts [g kg^−1^ d.m.].

Research Material	K	P	Mg	Ca
	**	**	**	**
Grain	4.85 ^b^ ± 0.24	3.32 ^b^ ± 0.35	1.15 ^b^ ± 0.06	0.43 ^b^ ± 0.10
Bran	8.50 ^c^ ± 1.55	7.66 ^c^ ± 0.27	3.72 ^c^ ± 0.22	1.02 ^c^ ± 0.19
Flour	2.57 ^a^ ± 0.17	1.82 ^a^ ± 0.15	0.38 ^a^ ± 0.05	0.25 ^a^ ± 0.06
Bread	2.87 ^a^ ± 0.16	1.91 ^a^ ± 0.17	0.40 ^a^ ± 0.03	0.31 ^ab^ ± 0.05

The data are presented as means of three farming systems with their respective standard deviations. Abbreviations: ** denotes significance, where different letters on the top of data ^a–c^ indicate significant differences at the α = 0.05 level between means according to Tukey’s test. K—potassium, P—phosphorus, Mg—magnesium, Ca—calcium.

**Table 4:** Micronutrients concentration in wheat grain and grain byproducts [mg kg^−1^ d.m.].

Research Material	Fe	Mn	Zn	Cu
	**	**	**	**
Grain	35.68 ^c^ ± 11.64	23.69 ^b^ ± 7.23	29.64 ^b^ ± 6.35	2.49 ^b^ ± 0.83
Bran	99.1 ^d^ ± 15.77	74.4 ^c^ ± 22.34	80.3 ^c^ ± 15.97	7.71 ^c^ ± 1.97
Flour	11.17 ^a^ ± 2.49	5.61 ^a^ ± 2.18	10.11 ^a^ ± 2.87	1.21 ^a^ ± 0.26
Bread	24.45 ^b^ ± 5.94	5.66 ^a^ ± 1.85	12.97 ^a^ ± 3.22	2.31 ^b^ ± 0.28

The data are presented as means of three farming systems. Abbreviations: ** denotes significance, where different letters on the top of data ^a–c^ indicate significant differences at the α = 0.05 level between means according to Tukey’s test. Fe—iron, Mn—manganese, Zn—zinc, Cu—copper.

## Data Availability

The original contributions presented in the study are included in the article/Supplementary Materials; further inquiries can be directed to the corresponding author.

## Supplementary Materials

The following supporting information can be downloaded at: https://www.mdpi.com/article/10.3390/plants14071003/s1, Table S1: Macro- [g kg^−1^ d.m.] and micronutrients [mg kg^−1^ d.m.] concentration in wheat grain; Table S2: Interaction effect of study factors on macro- and micronutrients concentration in the grain; Figure S1: Correlation between analyzed macro- and micronutrients in grain from CONV system; Figure S2: Correlation between analyzed macro- and micronutrients in grain from INT system; Figure S3: Correlation between analyzed macro- and micronutrients in grain from ORG system.

## Abbreviations

The following abbreviations are used in this manuscript:

ORG	Organic
INT	Integrated
CONV	Conventional
K	Potassium
P	Phosphorus
Mg	Magnesium
Ca	Calcium
Fe	Iron
Zn	Zinc
Mn	Manganese
Cu	Copper
d.m.	Dry matter
GR	Grain
BN	Bran
FR	Flour
BD	Bread
