# Supplementary material for: Mineral Concentration in Spring Wheat Grain Under Organic, Integrated, and Conventional Farming Systems and Their Alterations During Processing
Source: Plants (Basel). 2025 Mar 23;14(7):1003. doi: 10.3390/plants14071003 (PMC11990186; doi:10.3390/plants14071003)
Supplement: Supplementary file 1 [file plants-14-01003-s001.zip › plants-3500748-supplementary.pdf]

Article

Mineral Concentration in Spring Wheat Grain Under Organic, Integrated, and Conventional Farming Systems and Their Alterations During Processing

Katarzyna Wysocka [1,*], Grażyna Cacak-Pietrzak [1,*] and Tomasz Sosulski [2]

[1] Department of Food Technology and Assessment, Institute of Food Sciences, Warsaw University of Life Sciences (SGGW), Nowoursynowska 159C Street, 02-776 Warsaw, Poland; katarzyna_wysocka@sggw.edu.pl (K.W.); grazyna_cacak_pietrzak@sggw.edu.pl (G.C-P.)
[2] Department of Agricultural and Environmental Chemistry, Institute of Agriculture, Warsaw University of Life Sciences (SGGW), Nowoursynowska 159 Street, 02-776 Warsaw, Poland; tomasz_sosulski@sggw.edu.pl (T.S.)
* Correspondence: katarzyna_wysocka@sggw.edu.pl (K.W.); grazyna_cacak_pietrzak@sggw.edu.pl (G.C-P.)

Table and Figure Contents:

Table S1. Macro- [g kg^{-1} d.m.] and micronutrients [mg kg^{-1} d.m.] concentration in grain.

Table S2. Interaction effect of study factors on macro- and micronutrients concentration in grain.

Figure S1. Correlation between analyzed macro- and micronutrients in grain from CONV system.

Figure S2. Correlation between analyzed macro- and micronutrients in grain from INT system.

Figure S3. Correlation between analyzed macro- and micronutrients in grain from ORG system.

Table S1. Macro- [g kg^{-1} d.m.] and micronutrients [mg kg^{-1} d.m.] concentration in grain.

Cultivar	Farming system	Year	Macronutrients [g kg^{-1} d.m.]				Micronutrients [mg kg^{-1} d.m.]			
			K	P	Mg	Ca	Fe	Mn	Zn	Cu
Harenda	ORG	2019	4.63	3.54	1.14	0.44	30.99	21.23	26.81	3.42
Harenda	ORG	2020	5.08	3.59	1.17	0.40	26.51	17.80	31.11	3.58
Harenda	ORG	2021	4.65	3.49	1.28	0.46	36.07	16.93	31.60	3.74
Harenda	INT	2019	4.59	3.64	1.11	0.51	33.76	20.45	26.63	2.06
Harenda	INT	2020	4.90	3.07	1.13	0.26	31.11	34.56	35.68	2.72
Harenda	INT	2021	4.70	3.35	1.20	0.37	37.81	31.69	35.10	2.48
Harenda	CONV	2019	5.00	3.72	1.18	0.64	33.70	19.54	23.63	2.12
Harenda	CONV	2020	4.90	3.46	1.18	0.36	30.20	41.26	32.71	1.96
Harenda	CONV	2021	4.59	3.41	1.17	0.54	39.83	24.93	27.53	1.63
Kandela	ORG	2019	5.01	3.26	1.05	0.37	26.52	17.75	27.46	3.31
Kandela	ORG	2020	5.20	3.38	1.09	0.36	25.85	16.62	30.39	3.83
Kandela	ORG	2021	4.70	3.03	1.16	0.41	72.58	16.32	31.01	4.06
Kandela	INT	2019	4.48	3.41	1.11	0.44	31.72	22.32	22.60	2.28
Kandela	INT	2020	5.02	3.47	1.10	0.43	22.71	26.11	25.41	1.68
Kandela	INT	2021	5.10	3.22	1.24	0.47	54.57	25.18	27.58	2.74
Kandela	CONV	2019	5.12	3.89	1.30	0.62	29.27	13.44	22.29	2.15
Kandela	CONV	2020	5.04	3.25	1.14	0.39	21.71	32.02	27.84	1.42
Kandela	CONV	2021	4.31	2.62	1.09	0.43	55.91	30.75	28.90	1.49
Mandaryna	ORG	2019	4.43	2.48	1.11	0.39	25.48	18.92	24.47	3.06
Mandaryna	ORG	2020	5.19	3.12	1.18	0.36	23.34	18.17	28.27	4.18
Mandaryna	ORG	2021	4.56	2.91	1.22	0.33	26.38	15.87	28,02	2,08
Mandaryna	INT	2019	4.67	3.23	1.25	0.55	33.81	24.88	29.62	2.30
Mandaryna	INT	2020	5.14	3.30	1.13	0.43	23.40	34.05	27.67	1.89
Mandaryna	INT	2021	4.61	2.89	1.15	0.43	47.46	19.53	26.70	2,23
Mandaryna	CONV	2019	4.98	3.28	1.19	0.59	28.14	16.50	21.84	2.28
Mandaryna	CONV	2020	5.17	3.25	1.19	0.67	22.18	31.10	26.43	1.81
Mandaryna	CONV	2021	4.63	2.77	1.17	0.43	64.96	15.28	26.31	2.02
Serenada	ORG	2019	5.11	3.17	1.07	0.37	39.60	23.57	31.23	3.01
Serenada	ORG	2020	4.93	4.05	1.16	0.38	40.30	25.38	48.34	3.93
Serenada	ORG	2021	4.62	3.48	1.22	0.31	36.20	17.25	34.61	3.33
Serenada	INT	2019	4.91	3.02	1.07	0.45	37.34	21.28	23.88	1.09
Serenada	INT	2020	4.95	4.09	1.13	0.34	40.41	41.13	51.24	2.27
Serenada	INT	2021	4.81	3.45	1.20	0.42	42.90	25.92	37.17	2.32
Serenada	CONV	2019	5.13	3.29	1.11	0.39	38.54	21.54	21.59	1.32
Serenada	CONV	2020	4.93	3.66	1.13	0.33	32.53	32.91	34.88	1.99
Serenada	CONV	2021	4.81	3.46	1.16	0.38	40.85	20.60	30.34	2.01
	**mean**		4.85	3.32	1.15	0.43	35.80	23.69	29.64	2.49

Abbreviations: ORG–organic, INT–integrated, CONV–conventional, K–potassium, P–phosphorus, Mg–magnesium, Ca–calcium, Fe–iron, Mn–manganese, Zn–zinc, Cu–copper.

Table S2. Interaction effect of study factors on macro- and micronutrients concentration in the grain.

Interaction effect	Macronutrients				Micronutrients			
	K	P	Mg	Ca	Fe	Mn	Zn	Cu
Year x Cultivar	n.s.	**	n.s.	**	**	**	**	n.s.
Year x Farming system	n.s.	**	n.s.	**	n.s.	**	**	**
Cultivar x Farming system	n.s.	**	n.s.	**	n.s.	**	**	**
Year x Cultivar x Farming system	n.s.	**	**	**	**	**	**	**

Abbreviations: **–significant, n.s. –not significant, K–potassium, P–phosphorus, Mg–magnesium, Ca–calcium, Fe–iron, Zn–zinc, Mn–manganese, Cu–copper.

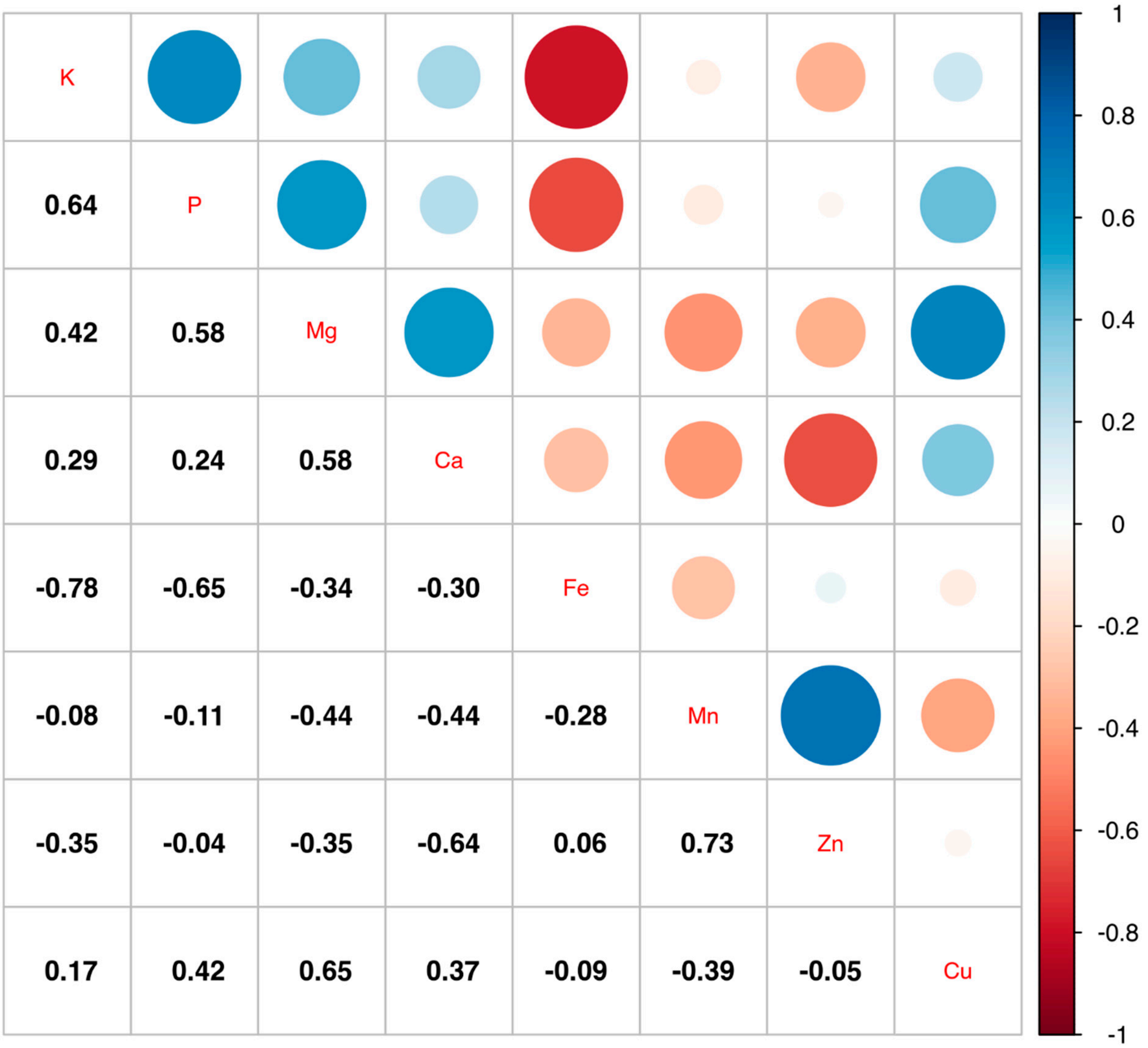

Figure S1. Correlation between analyzed macro- and micronutrients in grain from CONV system. The blue color indicates a positive correlation, the red one negative between the measured nutrients. Abbreviations: K–potassium, P–phosphorus, Mg–magnesium, Ca–calcium, Fe–iron, Mn–manganese, Zn–zinc, Cu–copper.

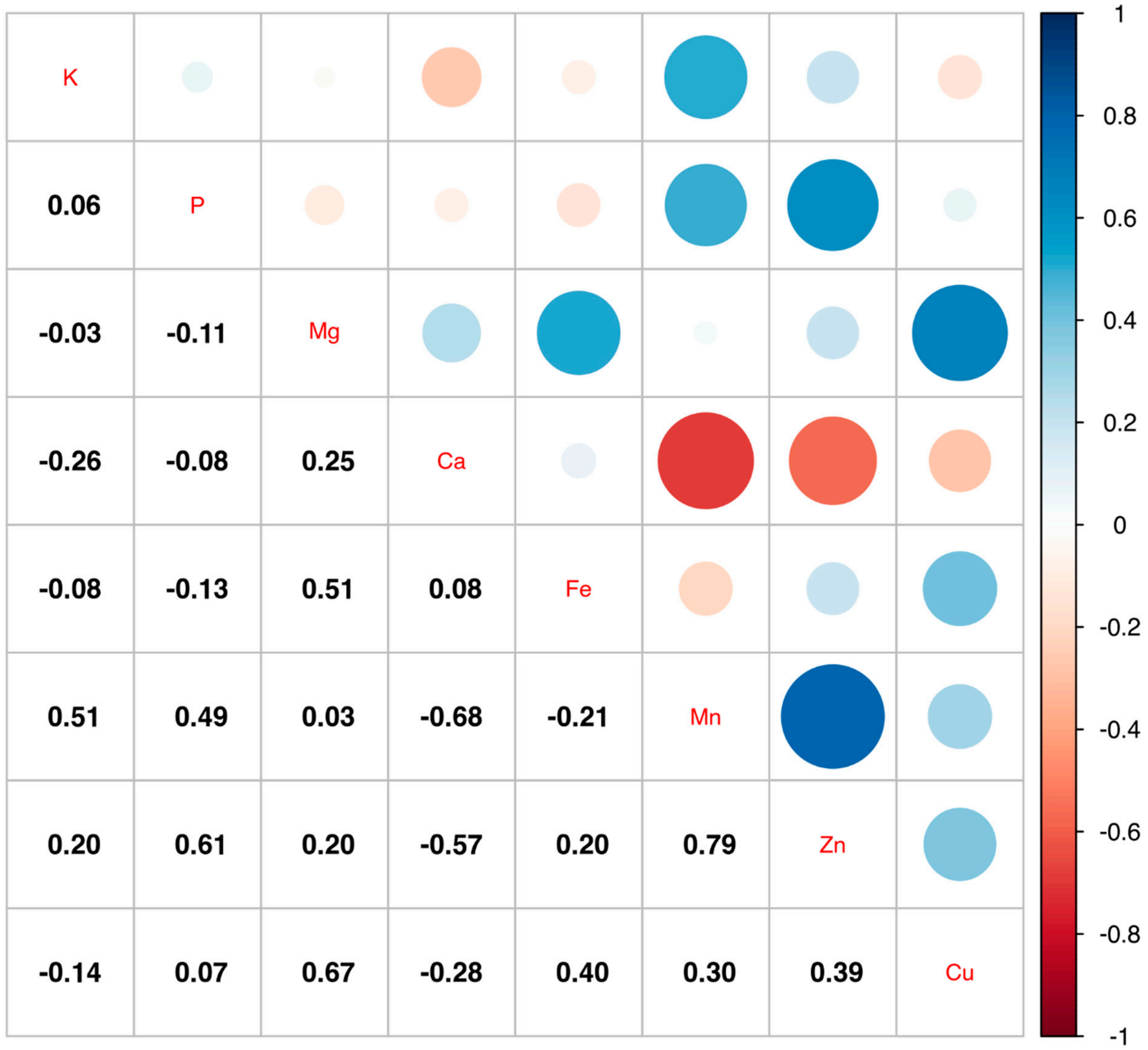

Figure S2. Correlation between analyzed macro- and micronutrients in grain from INT system. The blue color indicates a positive correlation, the red one negative between the measured nutrients. Abbreviations: K–potassium, P–phosphorus, Mg–magnesium, Ca–calcium, Fe–iron, Mn–manganese, Zn–zinc, Cu–copper.

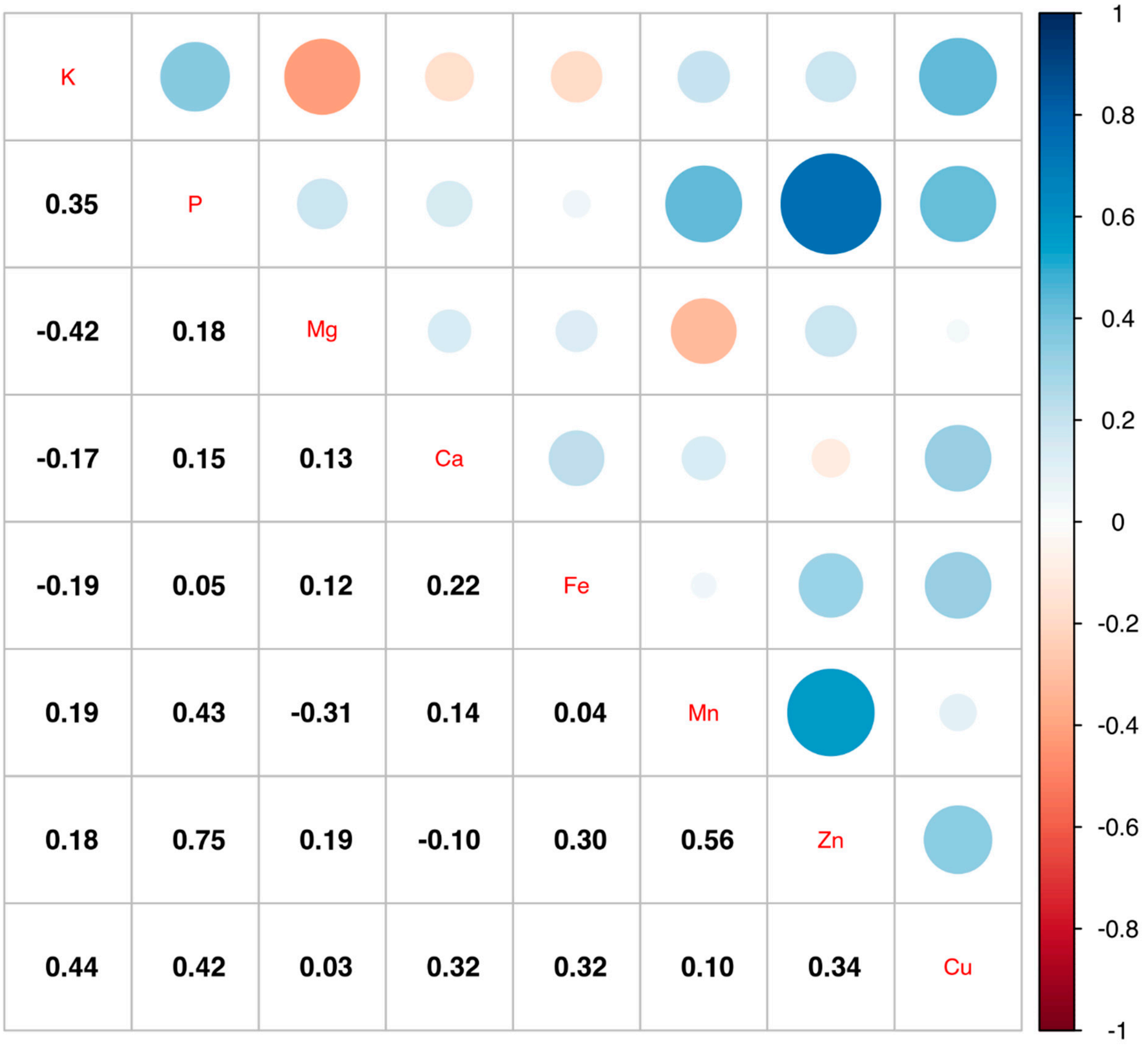

Figure S3. Correlation between analyzed macro- and micronutrients in grain from ORG system. The blue color indicates a positive correlation, the red one negative between the measured nutrients. Abbreviations: K–potassium, P–phosphorus, Mg–magnesium, Ca–calcium, Fe–iron, Mn–manganese, Zn–zinc, Cu–copper.
